# Exercise-Induced Rhabdomyolysis Causing Acute Kidney Injury: A Potential Threat to Gym Lovers

**DOI:** 10.7759/cureus.28046

**Published:** 2022-08-15

**Authors:** Ravi Kumar, Sanjay Kumar, Ankeet Kumar, Deepak Kumar, Vikash Kumar

**Affiliations:** 1 Internal Medicine, Jinnah Sindh Medical University, Karachi, PAK; 2 Internal Medicine, Liaquat University of Medical and Health Sciences, Jamshoro, PAK; 3 Internal Medicine, The Brooklyn Hospital Center, New York, USA

**Keywords:** exertional rhabdomyolysis in young adults, complications of rhabdomyolysis, exercise induce rhabdomyolysis causing acute kidney injury, rhabdomyolysis causing acute kidney injury, acute kidney injury

## Abstract

Rhabdomyolysis, by definition, means the breakdown of muscles. The common causes are trauma, immobility, illicit drug use, medications, toxins, infections, potassium imbalance, hypothyroid or hyperthyroid states, hypothermia or hyperthermia, and some congenital muscular dystrophy. Exercise or exertion-induced rhabdomyolysis is a very uncommon entity and potentially rising among young generations amid getting perfect body shape as influenced by social platforms. However, rhabdomyolysis can lead to lethal complications, most commonly acute kidney injury leading to dialysis, disseminated intravascular coagulation (DIC), and acute compartment syndrome. Here we report a case of exertion-induced rhabdomyolysis causing acute renal failure in a young patient who presented to the emergency room at The Kidney Center, Karachi, after exercising at the gym. The patient was subsequently treated with hemodialysis and was discharged after six days of hospital admission.

## Introduction

The term rhabdomyolysis is defined as the breakdown of striated muscles resulting from direct or indirect trauma to the muscle, which potentially releases its intracellular contents (myoglobin, creatine kinase {CK}, aldolase, and lactate dehydrogenase) in blood circulation resulting in devastating complications to body organs like an acute renal failure and disseminated intravascular coagulation (DIC) [1]. The exercise-induced rhabdomyolysis is caused after having heavy exercise, a sudden onset start of exercise, or exercise done for a prolonged time [1-3]. It is potentially seen and growing in large numbers in younger adults as influenced by social media platforms to have six packs abs as a body complexion [2,4]. In addition, people lacking proper knowledge or proven exercise in the gym and exercising beyond personal physical limits leads to an increased risk of rhabdomyolysis in these young adults. This gym-induced rhabdomyolysis potentially leads to rapid onset acute kidney injury that needs long-term stay at the hospital and aggressive treatment with dialysis.

## Case presentation

A 26-year-old male with no past medical or family history presented to the emergency department with complaints of decreased urine output, fatigue, and thigh and calf pain. As per the patient, he was in his usual state of health after three days of joining the gym and starting doing heavy exercise without a trainer; he started feeling fatigued and noticed brownish discoloration of urine along with decreased urine output on the next day. The patient also reported having bilateral posterior thigh and calf pain and stated that he could not bend on his knees. The pain was achy, sudden onset, 8/10 intensity, non-radiating, aggravated with movements, and no relieving factors. The patient denied fever, chills, nausea, vomiting, burning micturition, and abdominal or flank pain. The patient reported on further history that he does not use illicit drugs, anabolic steroids, alcohol, or any medication.

On presentation to the emergency department, the patient was alert and oriented. Physical examination showed a thin-built male with calf tenderness and no pedal edema; the rest of the general examination was unremarkable. Vitals signs were as follows; blood pressure (BP): 130/85, temperature: 37 °C or 98.6 F, pulse: 113 beats per minute, SpO_2_: 99%.

A bedside venous/arterial doppler ultrasound was negative for deep venous thrombosis. Blood investigations and urine analysis showed high levels of serum creatinine 12.65 mg/dl, blood urea nitrogen (BUN) 94 mg/dl, creatine phosphokinase (CPK) 87750 U/L, and lactate dehydrogenase (LDH) 787 U/L confirming the diagnosis of acute kidney injury due to exercise-induced rhabdomyolysis. Urine analysis was negative for protein and occult blood. Ultrasound of kidneys and bladder was normal. Arterial blood gasses (ABgs) showed metabolic acidosis with fully compensated repository alkalosis.

The patient was started with intravenous hydration with normal saline and sodium bicarbonate in the emergency department and admitted to the medical floor for further management. Results of laboratory workups on the day of admission are shown in Table 1.

**Table 1:** Laboratory testing results on the day of admission CBC: Complete blood count; ABG: Arterial blood gasses; CPK: Creatine phosphokinase; LDH: Lactate dehydrogenase

Labs Variable	Result Reading on Admission Day	Reference Range
Hemoglobin, g/dl	12.6	M: 13.5 - 17.5 F: 11.5 - 15.5
White Cell Count, 10^9^/L	10.41	4.0 - 10.0
Platelets Count, 10^9^/L	318	150 - 400
Creatinine, mg/dl	12.6	0.7 - 1.2
Creatinine Phosphokinase (CPK), U/L	87,750	M: 46 -171, F: 34 - 145
Blood Urea Nitrogen (BUN), mg/dl	94	06- 20
Lactate Dehydrogenase (LDH), Adult, U/L	787	125 - 220
Urea, mg/dl	201	10 - 50
Sodium (Na), M Eq/L	126	136 - 149
Potassium (K), M Eq/L	4.5	3.8 - 5.2
Chloride (Cl), M Eq/L	99	98 - 107
Bicarbonate (HCO_3_), M Eq/L	15	25 -29
Calcium (Adults), mg/dl	6.69	8.6 - 10.2
Phosphorus (Adults), mg/d	6.94	2.5 - 4.5
Albumin, g/dL	3.38	3.5 - 5.2
pH	7.41	7.35 - 7.45
PCO_2_, mmHg	24.1	35 – 45
PO_2_, mmHg	135.5	75 - 100
HCO_3_, mmol/l	15.6	22 - 24
SAT, %	99.2	95 - 98

The patient did not improve on intravenous hydration therapy as there was no significant urinary output for 24 hours and there was an increasing trend of creatinine. The patient was subsequently started on hemodialysis on the 2nd day of admission with clearance for 45 minutes and nil ultrafiltrate. Access was obtained through the internal jugular under aseptic measures. The patient showed good improvement with hemodialysis sessions and there was a decreasing trend noted in creatinine levels. A total of five hemodialysis sessions were done and the patient was discharged on the 6th day of hospital admission. The patient had a follow-up after two weeks of hospital discharge with all the labs, which were within normal range and the patient did not report any new symptoms. Creatinine, Urea, BUN, CPK, and LDH from days 1st to 3rd, 6th day, and follow-up after two weeks of discharge are shown in Table 2.

**Table 2:** Comparison of laboratory testing results obtained on admission day, during treatment, on the day of discharge, and at 2 weeks follow-up

Variables	Day1 (Hospital Admission)	Day 2	Day 3	Day 6 Hospital Discharge	Follow-up after 2 weeks of discharge
Creatinine, mg/dl	12.6	14.33	11.71	2.5	0.9
Urea, mg/dl	201	227	222	80	30
Blood Urea Nitrogen (BUN), mg/dl	94	106	104	28	12
Creatinine Phosphokinase (CPK), U/L	87,750	89,201	43,850	2209	130
Lactate Dehydrogenase (LDH), Adult, U/L	787	572	320	198	129

## Discussion

The exercise-induced rhabdomyolysis is a sub-division of exertion-induced rhabdomyolysis, saying that exercise-induced occurs in those who have a high risk of developing factors that include exercise done by an untrained person, certain types of exercise (eccentric) muscle lengthening, and downhill exercises, or exercise done for prolong time beyond personal capacity [3-6]. Along with it, predisposing factors can contribute to developing it, which include dehydration, humid and hot temperatures [7-8], certain genetic mutations [9], and alcohol and heroin use [10,11]. Since our patient was untrained, being dehydrated and exercising beyond personal capacity were contributing factors. The incidence of exercise-induced rhabdomyolysis causing acute kidney injury is higher in males than females and this unusual trend is because males don't prefer to have a personal professional trainer at the gym and also due to uneven distribution of skeletal muscle and fat being more in males as compared to females [2]. Weisenthal et al. did a survey in 2014 among CrossFit participants in the united states to see muscle injury & the study results showed that males developed more muscle injuries than females because of fewer trainers obtained by males [12].

The exact mechanism of exercise inducing rhabdomyolysis has been postulated with a decrease or depletion of adenosine triphosphate (ATP) during heavy exercises for a prolonged time. Low levels of ATP lead to shutting down calcium channels and leading to increased levels of intracellular calcium, which activates protease and phospholipase A2, which break down the intracellular structures leading to cell death [3,4]. The incidence of acute renal failure in patients with exercise-induced rhabdomyolysis is around 10-30% [3-4,13]. The primary cause is the release of muscle's intracellular contents, mostly myoglobin, which is freely filtrated through the glomerular basement membrane and accumulated into tubules, leading to acute kidney injury. Also, hypovolemia tends to cause renal vaso-constriction, which further activates the renin-angiotensin-aldosterone system leading to renal azotemia [5]. 

The presentation of exercise induces rhabdomyolysis causing acute kidney injury, mostly seen one to two days after heavy exercise. The most common triad of symptoms is myalgias, muddy brown urine, and decreased urine output. Most patients will provide a history of recently joining the gym and starting heavy weight-bearing exercises without a trainer. Therefore, a presumed diagnosis can be made through these symptoms and history. Serum creatinine and urea are elevated, indicating acute renal failure, and serum creatinine kinase (CK) levels five times higher than normal upper limits have been indicted by most physicians to diagnose exercise-induced rhabdomyolysis [1,14]. Serum CK is more reliable than serum myoglobin to see the intensity of muscle damage because of the slow degradation and prolonged sustainability of serum CK than myoglobin [5]. The treatment usually starts with intravenous hydration to achieve urine output, but most of these patients tend to benefit from dialysis and the course seems benign. However, our patient's initial hydration did not improve renal function and he eventually benefited from hemodialysis and was discharged on the 6th day of hospital admission. 

## Conclusions

The incidence of exercise-induced rhabdomyolysis is rising among the young generations because of social influence to have a perfect body shape and too less information is available about this complication. Therefore, the main aim of writing this case report is to inform and aware health care physicians to educate the general population regarding prevention measures like warm-up exercises. Furthermore, having a professional trainer and rehydration can decrease the chances of developing this complication.

## References

[1] Peng F, Lin X, Sun LZ (2019). Exertional rhabdomyolysis in a 21-year-old healthy man resulting from lower extremity training: a case report. Medicine (Baltimore).

[2] Knafl EG, Hughes JA, Dimeski G, Eley R (2018). Rhabdomyolysis: patterns, circumstances, and outcomes of patients presenting to the emergency department. Ochsner J.

[3] Al Badi A, Al Rasbi S, Alalawi AM (2020). Exercise-induced rhabdomyolysis: a case report and literature review. Cureus.

[4] Kim J, Lee J, Kim S, Ryu HY, Cha KS, Sung DJ (2016). Exercise-induced rhabdomyolysis mechanisms and prevention: a literature review. J Sport Health Sci.

[5] Vanholder R, Sever MS, Erek E, Lameire N (2000). Rhabdomyolysis. J Am Soc Nephrol.

[6] Dekeyser B, Schwagten V, Beaucourt L (2009). Severe rhabdomyolysis after recreational training. Emerg Med J.

[7] Schwaber MJ, Liss HP, Steiner I, Brezis M (1994). Hazard of sauna use after strenuous exercise. Ann Intern Med.

[8] Furman J (2015). When exercise causes exertional rhabdomyolysis. JAAPA.

[9] Scalco RS, Gardiner AR, Pitceathly RD (2015). Rhabdomyolysis: a genetic perspective. Orphanet J Rare Dis.

[10] Vitris M, Saissy JM, Gohard R, Raux O, Diatta B, Kemps J (1991). Heroin-induced acute rhabdomyolysis (Article in French). Dakar Med.

[11] Htet Z (2018). Alcohol-induced rhabdomyolysis: a disease with potential pitfalls. Acute Med.

[12] Weisenthal BM, Beck CA, Maloney MD, DeHaven KE, Giordano BD (2014). Injury rate and patterns among CrossFit athletes. Orthop J Sports Med.

[13] Huerta-Alardín AL, Varon J, Marik PE (2005). Bench-to-bedside review: rhabdomyolysis -- an overview for clinicians. Crit Care.

[14] Chavez LO, Leon M, Einav S, Varon J (2016). Beyond muscle destruction: a systematic review of rhabdomyolysis for clinical practice. Crit Care.

